# Using Deep Learning to Automate Orangutan Nest Detections on Aerial Images Collected With Drones

**DOI:** 10.1002/ajp.70100

**Published:** 2025-12-07

**Authors:** Serge Wich, Marc Ancrenaz, Benoit Goossens, Molly Hennekam, Sol Milne, David Burslem, Cheryl Knott, Julien Martin, Paul Fergus

**Affiliations:** ^1^ School of Biological and Environmental Sciences Liverpool John Moores University Liverpool UK; ^2^ HUTAN Sandakan Malaysia; ^3^ Borneo Futures Bandar Seri Begawan Brunei; ^4^ c/o Sabah Wildlife Department Danau Girang Field Centre, Wisma MUIS Kota Kinabalu Sabah Malaysia; ^5^ Cardiff School of Biosciences, Organisms and Environment Division Cardiff University Cardiff UK; ^6^ Sabah Wildlife Department Wisma MUIS Kota Kinabalu Malaysia; ^7^ Uncrewed Research Aircraft Facility, Division of Research and Innovation The University of Adelaide Adelaide Australia; ^8^ Wolf Fish Ltd Brecon Wales UK; ^9^ Interdisciplinary Institute and School of Biological Sciences University of Aberdeen Aberdeen UK; ^10^ Department of Anthropology, Department of Biology Boston University Boston Massachusetts USA; ^11^ Department of Biology University of Ottawa Ottawa Ontario Canada; ^12^ School of Computer Science and Mathematics Liverpool John Moores University Liverpool UK

**Keywords:** great apes, Indonesia, line transects, Malaysia, monitoring

## Abstract

Traditional orangutan distribution and density monitoring requires costly line transect methods on the ground to detect their nests. Recently researchers have started to use unoccupied aerial vehicles, hereafter referred to as drones, to collect such data faster. However, manually inspecting the images acquired by the drone is time‐consuming and hence costly. This study explored a deep learning method for the automated detection of orangutan nests in drone‐captured aerial images, which can significantly improve the efficiency of orangutan monitoring efforts. The YOLO v10 model was trained using 868 images containing 1568 annotated orangutan nests collected from sites in Sabah, Malaysia, and Sumatra, Indonesia. Images were captured using multirotor and fixed‐wing drones at varying altitudes. The model was trained using a transfer learning approach and achieved a mean Average Precision (mAP) of 0.831. The model was subsequently tested on two independent data sets with results showing a precision of 0.98 and recall of 0.88 for a multirotor drone and precision of 0.98 and a recall of 0.71 for a fixed‐wing drone which has the benefit of being able to have longer duration flights. The high precision values indicate the model's accuracy in identifying true nest locations, while the recall values demonstrate its ability to detect a significant portion of the nests present in the images. The study highlights how using drones for data collection can reduce survey times compared to ground surveys, and the automation of nest detection further enhances the efficiency of drone surveys. However, the model's recall, especially for fixed‐wing drone data, could be improved to ensure accurate population trend analyses. Further research should focus on expanding training data sets and refining models to account for different camera systems and environmental conditions.

## Summary

- This study developed a deep learning approach for automated orangutan nest detection in drone‐captured images, significantly improving the efficiency of population monitoring.
- A YOLO v10 model trained on 868 images achieved high precision (0.98) but varying recall (0.88 for multirotor, 0.71 for fixed‐wing drones), highlighting the need for further improvements in detection consistency.
- Automating nest detection reduces survey time and costs compared to ground‐based methods, but future work should refine the model to enhance recall and adapt to diverse environmental conditions.

## Graphical abstract

Graphical abstract of the deep learning study to detect orangutan nests on images acquired by drones.

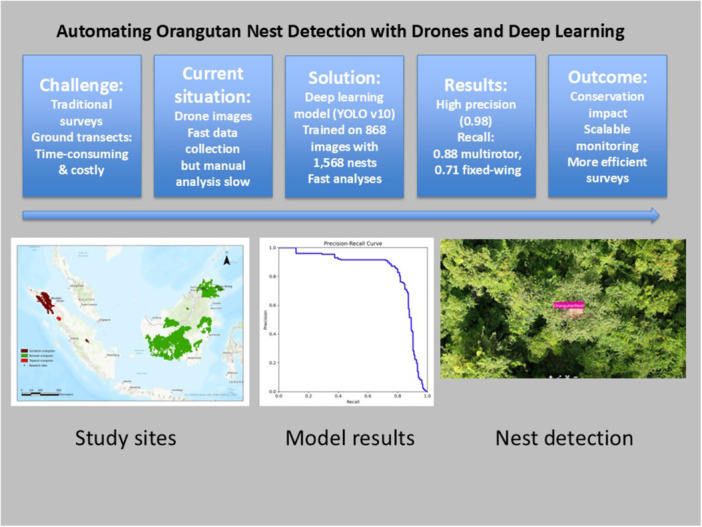

## 1 Introduction

Orangutans (*Pongo* sp.) are the only great ape species occurring in Southeast Asia, where they are found on the islands of Borneo and Sumatra. Historically, their distribution reached as far north as southern China, and as far south as the Indonesian island of Java (Bacon and Long 2001; Hooijer 1948; von Koeningswald 1982; Jablonski et al. 2000). Recent genetic studies have determined there are three extant species (Nater et al. 2017) with two of these occurring on Sumatra (Sumatran and Tapanuli orangutan, *Pongo abelii* and *Pongo tapanuliensis*, respectively) and one on Borneo (Bornean orangutan, *Pongo pygmaeus*). The numbers of all three orangutan species have dramatically decreased (by more than 80%) over the past three orangutan generations (~75 years) (Nowak et al. 2017; Ancrenaz et al. 2016; Singleton et al. 2016). As a result of this, all three species are listed as “Critically Endangered” on the IUCN Red List of Threatened Species (Nowak et al. 2017; Ancrenaz et al. 2016; Singleton et al. 2016).

The main threats to the continued survival of orangutans are forest loss, forest degradation, forest fragmentation, and illegal killing (Sabah Wildlife Department 2011; Voigt et al. 2018; Spehar et al. 2018; Santika et al. 2017; Wich, Singleton, et al. 2016). Besides populations being reduced by large‐scale habitat loss and hunting (Rijksen and Meijaard 1999; Meijaard, Welsh, et al. 2010; Meijaard, Buchori, et al. 2011; Meijaard, Mengersen, et al. 2011), the orangutan's unique life history makes the species even more vulnerable to extinction (Marshall et al. 2009). This is mostly a result of their large body size (Harvey et al. 1987) and their extremely slow reproductive rate. Female wild orangutans give birth to a single infant only once every 6–9 years (Galdikas and Wood 1990; Wich et al. 2004, 2009), the longest inter‐birth interval of any mammal. Further adding to their vulnerability is the fact that they live at very low densities which depend on their habitat type (0.01–7 individuals/km^2^ (Voigt et al. 2018); Wich, Singleton, et al. 2016). Orangutans also utilize extensive home ranges (up to over 10,000 ha for flanged males (Singleton et al. 2009; Singleton and van Schaik 2001)) meaning they need very large areas to exist in viable populations (Singleton et al. 2009). In addition, orangutans in Borneo are restricted almost entirely to remaining lowland forests, being generally rare or absent above 500 m above sea level (asl) (Wich et al. 2008). This limited distribution results in an overlap between the forests they need to survive and the areas that are most vulnerable to conversion to agriculture, pulp and paper plantations, and other land covers that replace forests (Voigt et al. 2018). In Sumatra, the altitudinal range of orangutans extends to, at least, 1500m asl (Wich, Singleton, et al. 2016), but densities are higher at lower altitudes and primarily situated in peat swamp forests that are being converted for agriculture (Wich, Singleton, et al. 2016; Wich et al. 2011). Orangutans increasingly use and are found in multiple‐use forests, including tree and oil palm plantations and other nonforest types of habitat, which also requires that we use different approaches to monitoring and managing these populations (Meijaard, Albar, et al. 2010; Ancrenaz et al. 2014, 2021).

There is thus a great need to monitor orangutans at large spatial scales and to obtain data for trend analyses so that populations can be better managed. Traditionally, orangutan abundance is determined by conducting linear transects and counting their nests (van Schaik et al. 1995; Buij et al. 2003). Orangutans usually build sleeping platforms (termed nests) in trees once or twice a day (Prasetyo et al. 2009). Nests are often categorized into 4–5 classes depending on their decay stage (Prasetyo et al. 2009) and the proportion of nests detected by human observers tends to vary between these decay stages and observer skills (Wich and Boyko 2011). Due to heterogeneity in environmental conditions, these nests have varying decay times which can lead to large confidence intervals in the orangutan densities derived from nest densities (van Schaik et al. 1995; Marshall and Meijaard 2009). In addition nest counts along transects have the assumption that all nests above the transect line are detected as well as the challenge that the proportion of nest builders, the number of nests built per day, and the nest decay rate vary between populations and would ideally be estimated at each survey area (Marshall and Meijaard 2009; Spehar et al. 2010; Mathewson et al. 2008). The large confidence intervals for nest surveys have led to tests for potential alternative methods, such as detecting orangutans directly with thermal infrared sensors (Burke et al. 2019) or suggestions to use nest counts for determining absence/presence and some measure of population trends (Wich, Dellatore, et al. 2016).

Because of the relatively large areas of orangutan distribution, as well as the difficulty of traversing the often mountainous or peat swamp terrain in which they occur, line transect surveys are expensive and time‐consuming (Wich and Koh 2018) and have left large parts of the orangutan range unsurveyed. As a result, researchers have explored alternatives, for example, using human observers to detect orangutan nests from helicopters and using those data to derive abundance estimates (Ancrenaz, Goossens, et al. 2004; Ancrenaz et al. 2005). The benefit of using crewed helicopters is that large areas can be covered in a short time, but helicopters have limited availability, are costly, require experienced nest spotters, and bring an inevitable risk of accidents (Sasse 2003). Therefore, researchers have started to use drones to monitor nests of orangutans (Wich, Dellatore, et al. 2016; Milne et al. 2021) as well as those of chimpanzees (*Pan troglodytes*) (Bonnin et al. 2018). With this method, fewer orangutan nests are detected than with ground‐based methods, but the aerial and ground counts correlate significantly (Wich, Dellatore, et al. 2016) and orangutan density can be determined from aerial nest counts (Milne et al. 2021). As a result, several projects are now using drones to monitor orangutan nests (Wich, Dellatore, et al. 2016, as well as the projects of the authors of this study). However, the time and cost reductions that can be made by using drones for data collection are, at present, diminished by the large numbers of images that must be inspected visually by human observers to find the nests in the images. This manual procedure is extremely time‐consuming, costly and reduces the net benefit of aerial over ground‐based surveys (Wich and Koh 2018). To reduce the time spent by humans on visually inspecting images for animals or their signs, there have been efforts to automate the detection process through machine learning approaches such as YOLO (You Only Look Once) and Faster‐RCNN (Region Based Convolutional Neural Networks) (Ren et al. 2016) for a variety of animal species (Wich and Koh 2018; Lamba et al. 2019; Chalmers et al. 2021; Bondi et al. 2019; Corcoran et al. 2019). At present, there is no freely available automated detection model for orangutan nest detection that generalizes for different areas where orangutans occur, despite the potential to vastly reduce or eliminate the time taken in human nest classification tasks and to help nonexperts to detect nests in images.

The aims of this study were therefore to develop, train, validate, and test a deep learning method to automatically detect orangutan nests in photographs captured via drone surveys.

## 2 Methods

### 2.1 Study Sites and Drones

**Figure 1:**
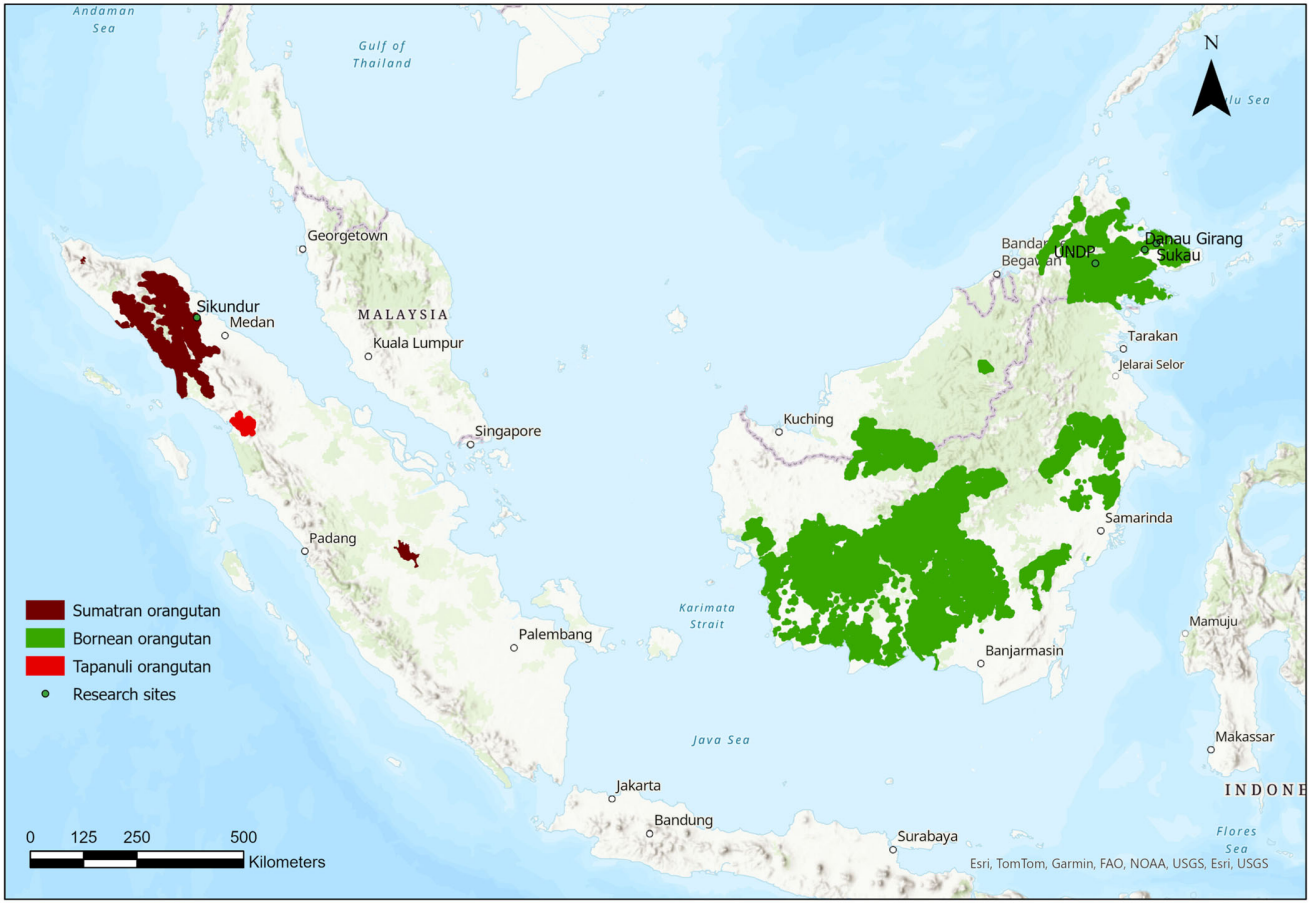
Map indicating the distributions of the three orangutan species and the locations where data have been collected.

Drone images used for training deep learning methods were collected at three sites in Sabah, Malaysia, with multirotor drones flying at varying heights between 2018 and 2024 and with a fixed‐wing drone at one site on Sumatra in 2012 (Figure 1). The sites in Sabah were the Hutan Intensive research study site, located in Lot 2 of the Lower Kinabatangan Wildlife Sanctuary (5° 32′ N, 118° 17′ E, (Ancrenaz, Calaque, et al. 2004)) near the village of Sukau and Danau Girang Field Centre (5° 24′ N, 118° 2′ E) along the Kinabatangan river (Lynn and Jumail 2021), and the UNDP‐GEF project area near Luasong (5° 6′ N, 116° 59′ E, (Milne et al. 2021)). The sites consisted of selectively logged and unlogged forest. A DJI Mavic 3E and DJI Phantom 4 Pro were flown between 50 and 100 m above ground level for data collection in Sabah. These are both small multirotor drones (Mavic 3E: 915 g, 20MP; Phantom 4 Pro: Weight 1338 g, camera sensor 20MP). In Sumatra, data were collected at the Sikundur site (3° 58′ N, 98° 05′ E, (Knop et al. 2004)), which consisted of selectively logged forest, with a Raptor Fixed Wing drone (weight ~2000 g, wingspan 1800 mm) with a Canon Powershot SX230 HS (camera sensor 12MP) that was flown between 80 and 120 m above ground level. All sites were either selectively logged or unlogged forest with closed canopies. A total of 868 images with 1568 nests were used to train the deep learning model. Fieldwork complied with the Codes for Best Practices in Field Primatology as well as National Regulations and the appropriate approval of the relevant institutes that the authors are affiliated with.

### 2.2 Deep Learning

The data set comprised images representing a single class, specifically orangutan nests, which were meticulously annotated by one observer (SAW) following the Pascal VOC format. Each image was uniformly segmented to a fixed resolution of 1824 × 1026 pixels to optimize model training performance. Following the annotation process, the data set was composed of 868 individual PNG files, with a total of 1568 annotations associated with the target class. See Supplementary Electronic Material for examples of annotated images and the whole workflow. The data set was further processed and converted into the YOLO v10 format using a custom Python script (Fergus et al. 2024). This script also facilitated the random partitioning of the images into training, testing, and validation subsets, ensuring an 80% allocation to the training set, 10% to the test set, and 10% to the validation set. This partitioning was used because a single‐class model was used and as such 1200 images for training, and a 150 for validation and testing each is sufficient to provide each of the three data sets a large enough number to be representative of the overall data. The partitioning was conducted based on the tagged labels to maintain consistency across the subsets (Fergus et al. 2024).

### 2.3 Model Selection

For modeling, YOLO 10x was used to perform both object detection and classification in a single stage. This approach allows the model to predict bounding box locations and class probabilities directly from the input image in a single pass through the YOLO network, leading to significantly faster detection without sacrificing accuracy. YOLO 10x eliminates the need for a separate region proposal network by dividing the image into a grid and predicting bounding boxes, confidence scores, and class probabilities for each grid cell. The model refines these predictions by minimizing a unified loss function that integrates both localization and classification. This single‐stage approach streamlines the detection process, enabling real‐time performance with enhanced computational efficiency. A transfer learning approach was used as it plays a crucial role in the effectiveness of YOLO v10 by enabling the adaptation of a pretrained model to detect and classify new objects of interest (Fergus et al. 2024). This method is particularly valuable when working with smaller data sets, such as in our case, as training convolutional neural networks (CNNs) from scratch on limited data often results in suboptimal performance due to reduced feature diversity and insufficient data variance and is thus not recommended. By leveraging a robust model that has been pretrained on a large‐scale data set, such as the COCO (Common Objects in Context) data set (containing 80 object categories) (Lin et al. 2014), transfer learning mitigates the challenges of limited sample size by utilizing already learned feature representations. This reduces the amount of data required for training while still achieving high accuracy (Fergus et al. 2024).

### 2.4 Modeling

The model was trained on an HP ProLiant ML 350 Gen. 9 server, equipped with dual Intel Xeon E5‐2640 v4 processors and 768 GB of RAM. To support high‐performance computations, the system was augmented with a GPU stack consisting of 8 Nvidia Quadro A6000 graphics cards, providing a cumulative 384 GB of GDDR6 memory. The training pipeline utilized PyTorch 2.01 in conjunction with CUDA 11.8 for optimized GPU acceleration. Key hyperparameters included a batch size of 32, an image size of 640, and a learning rate of 0.01, with the model trained across 10 epochs (one complete pass through the training data set during model training). Additional settings, such as Automatic Mixed Precision (AMP) and augmentation techniques like horizontal flipping and mosaic augmentation, were employed to enhance training efficiency and model generalization.

### 2.5 Data Sets for Model Tests

The model was tested with two data sets that were not used in the training but were similar in resolution. Because the original images from the drone flights in Sabah and Sumatra had a higher resolution (Sabah: 4864 × 3648, 3840 × 2160, and Sumatra: 4000 × 3000) than the Conservation AI YOLO object detection models (Chalmers et al. 2021; Fergus et al. 2024; Westworth et al. 2022; Doull et al. 2021) can handle, the images were segmented into 1824 × 1026 pixel segments. The first test data set was obtained in the HUTAN (Sukau, Sabah) study site during 2023 with a DJI Mavic 3E drone and consisted of image segments containing 65 nests. The second data set containing image segments with 214 nests was obtained at the same site in 2015 using a fixed‐wing drone and a Canon S100 camera. These image segments were subsequently uploaded to orangutan nest model which is hosted within the Conservation AI client (downloadable from https://www.conservationai.co.uk/) with a detection probability level set at 0.3 as this represented a good trade‐off between true and false positives. Data availability on request.

### 2.6 Evaluation Metrics

The model was evaluated using precision, recall, and loss (Fergus et al. 2023). Precision was used to measure the proportion of true‐positive detections that the model makes out of all detections: Precision = True Positives/(True Positives + False Positives). Recall was used to measure the proportion of positive detections detected by the model of all positives in the data set: Recall = True Positives/(True Positives + False Negatives). Loss measures how far the model's predictions are from the ground truth (Fergus et al. 2023).

## 3 Results

### 3.1 Training

**Figure 2:**
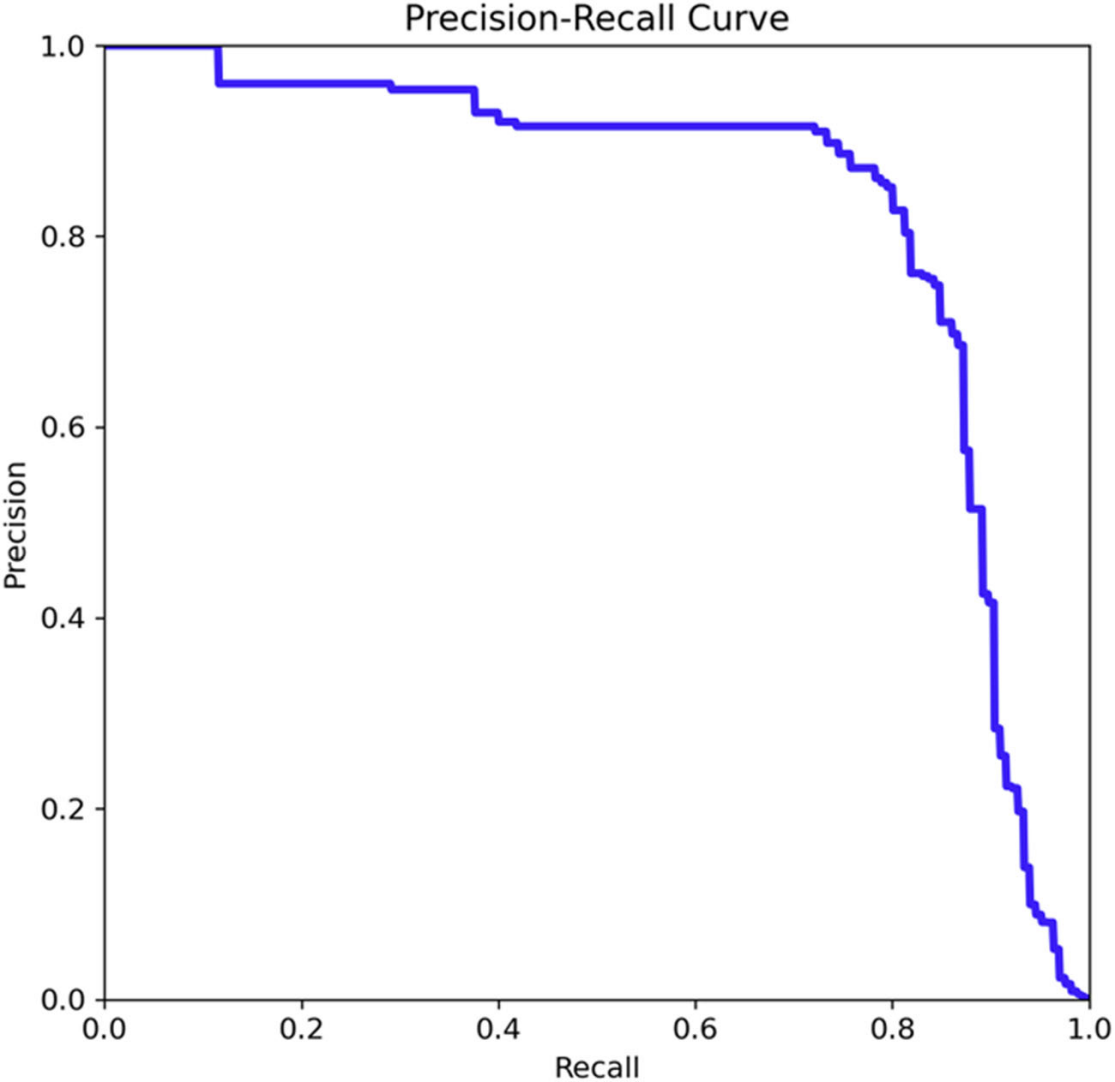
PR curve that indicates the relationship between precision and recall.

**Figure 3:**
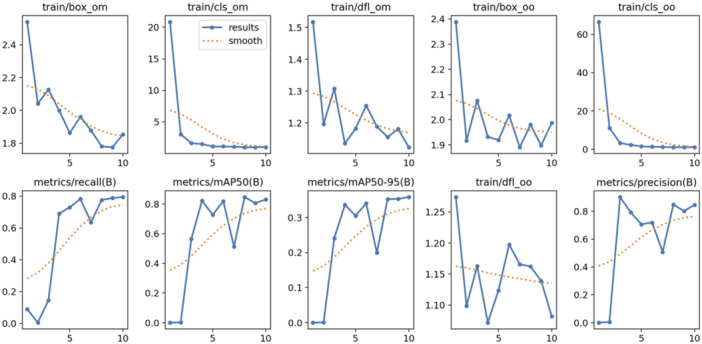
Training loss and performance metric trends. The training metrics for the object detection model are provided for 10 epochs. Train/box_om, train/cls_om, and train/dfl_om represent the loss components for bounding box regression, classification, and distribution focal loss for the object mask, respectively. Train/box_oo, train/cls_oo, and train/dfl_oo show the corresponding losses for object‐only predictions. The metrics recall(B), mAP50(B), and mAP50‐95(B) denote the recall, mean average precision at 50% IoU (Intersection over Union), and mean average precision across IoUs (50%–95%) for the validation set (B). Metrics/precision(B) refers to the precision on the same validation set. The solid blue line represents the raw metrics/loss values, while the dashed orange line indicates smoothed trends for better visualization.

The Precision‐Recall curve (Figure 2) indicates a strong mean Average Precision (mAP) of 0.831 at an IoU (Intersection over Union) threshold of 0.5, demonstrating that the model achieves high precision with minimal false positives across most recall levels. However, a slight decline in precision is observed as recall approaches 0.8. Figure 3 tracks the training loss and evaluation metrics over 10 epochs, where the box loss, class loss, and distribution focal loss all steadily decrease, indicating improved model performance in object localization and classification. The recall increases over time, reaching 0.8, while the mAP@50 stabilizes around 0.8, and mAP@50–95, a more stringent metric, converges around 0.3. Precision remains consistently high, reflecting the model's capability to reduce false positives. Overall, the results suggest that the YOLO v10 model effectively detects orangutan nests with a favorable balance between precision and recall.

### 3.2 Inference

#### 3.2.1 Model Test With Independent Data

Precision and recall for the first set of test nest images obtained with the DJI multirotor were 0.98 and 0.88, respectively. For the second set of test images obtained with the fixed‐wing drone, the precision and recall were 0.98 and 0.71, respectively.

## 4 Discussion

The study showcases the application of a deep learning model using the YOLO v10 algorithm for automated orangutan nest detection from drone imagery.

To evaluate the model's performance, the model (available on http://www.conservationai.co.uk after registration) was tested on two independent data sets collected using different drone platforms (a multirotor and fixed wing) and camera systems in two different forest areas. The results showed high precision and recall, demonstrating the model's ability to generalize to new environments and data sources. The high precision suggests that the model can accurately identify true nest locations and does not erroneously classify objects that are not nests as nests, while the high recall indicates that it successfully detects a significant proportion of the nests present in the imagery. However, particularly for the images obtained from the fixed‐wing drone the recall would ideally increase so that the models miss a smaller proportion of the true nests as the missed nest proportion from the model is higher than the proportion of nests missed by ground teams when monitoring nests which was only 0.02 by an experienced team of observers during ground surveys (Wich and Boyko 2011). The missed proportions by the model on the test data sets were 0.12 and 0.29, respectively. Nonetheless, these results underscore the robustness and effectiveness of the deep learning model, showing its potential for real‐world applications in orangutan conservation efforts despite the lower recall for fixed‐wing surveys, which could be due to a smaller number of annotated nests from fixed‐wing drones in the training data set than from multirotor drones. However, it is important to note that to determine population trends, the recall of models needs to be as high as possible so that analyses that compare the results of repeat surveys are able to detect small changes in population size. This condition is crucial for areas in which time‐series data are being collected or are planned to be collected. This issue is not unique to orangutan nest surveys with drones but also exists in ground nest counts that have large confidence intervals (Wich, Singleton, et al. 2016; Wich and Boyko 2011; Wich et al. 2019). Unfortunately, we were not able to directly compare the confidence intervals from ground surveys to those from drones as there has been relatively little work conducted on how many nests ground surveys miss and thus what their actual recall is (Wich and Boyko 2011; van Schaik et al. 2005). During this study, we carefully examined all images to ensure that only orangutan nest images were used and not images that contained nests of other species, such as raptors, sunbears, or squirrels. Future models would ideally include those classes as well.

Great ape data collection with drones reduces the time to survey areas compared to ground surveys (Wich and Koh 2018; Wich et al. 2023). In the case of orangutan nest surveys, a ground survey walk takes ~1 km/h by one person, whereas a drone can fly 18 km of transects during an hour. But those same studies indicated that the human image analyst time to check thousands of images for nests reduced the time gains made during the data collection part of the survey. An hour of image collection with a photo obtained every 2 s would generate 1800 images/h. A trained human image analyst would need ~1 min/image to look for nests, which would then take up to 30 h of time for a human analyst to check 1 h of drone flight. The automation of nest detection, as shown in this survey, can further increase the efficiency of drone surveys, as 1800 images can go through the machine learning pipeline in less than an hour, while the research can focus on something else. To really reach its full potential, models would ideally be further refined to increase recall. This will require larger data sets than available at present as well as models that deal well with data obtained from different camera systems at different heights. Model performance heavily depends on the quality and diversity of training data. Models trained on limited or biased data sets may struggle to generalize across new environments or conditions. Additionally, variations in nest appearance due to environmental conditions or nest age can affect detection accuracy. To enhance model robustness, future research should focus on expanding training data sets to include diverse nest ages and environmental conditions. Ideally, all surveys would be conducted using similar camera systems and fly at the same height above the canopy level. However, the ongoing development of drones by manufacturers will lead to researchers using different drone systems to meet differing environmental requirements. For example, in some settings, multirotor drones might be the only option (e.g., lack of fixed‐wing drone landing sites), whereas in others fixed‐wing drone systems might be used (e.g., the need to cover large areas). This difference is important because multirotor and fixed‐wing drones may deploy different camera systems, and fixed‐wing systems fly faster compared to multirotor drones. Flying speed is likely not to be of influence on image quality as long as there is no motion blur (Krzysztof 2011). Additionally, recent developments in vertical take‐off and landing (VTOL) or powered‐lift drones offer another alternative (covering large areas and with increased flexibility in landing sites), contributing to further variation in future data sets.

In conclusion, this study demonstrates that deep learning can support orangutan conservation by enabling automated nest detection. Further research is required to reduce recall, which would help to further increase the usage of these models for orangutan conservation.

## Author Contributions

**Serge Wich:** conceptualization (equal), funding acquisition (equal), writing – review and editing (equal). **Marc Ancrenaz:** conceptualization (equal), funding acquisition (equal), writing – review and editing (equal). **Benoit Goossens:** conceptualization (equal), funding acquisition (equal), writing – review and editing (equal). **Molly Hennekam:** conceptualization (equal), funding acquisition (equal), investigation (equal), writing – review and editing (equal). **Sol Milne:** conceptualization (equal), funding acquisition (equal), investigation (equal), writing – review and editing (equal). **David Burslem:** conceptualization (equal), funding acquisition (equal), writing – review and editing (equal). **Cheryl Knott:** conceptualization (equal), funding acquisition (equal), writing – review and editing (equal). **Julien Martin:** conceptualization (equal), funding acquisition (equal), writing – review and editing (equal). **Paul Fergus:** conceptualization (equal), formal analysis (equal), funding acquisition (equal), software (equal), writing – original draft (equal).

## Ethics Statement

The authors have nothing to report.

## Data Availability

Data availability on request.
